# Cumulative Lifestyle Profiles and Osteoporotic Fracture Risk in Older Women: A Nationwide Korean Cohort Study

**DOI:** 10.3390/healthcare14172812

**Published:** 2026-09-02

**Authors:** Dojoon Park, Hae-Seok Koh, Seokwon Jeong, Ju-Yeong Park, Kyungdo Han, Youn-Ho Choi

**Affiliations:** 1 Department of Orthopedic Surgery, St. Vincent’s Hospital, College of Medicine, The Catholic University of Korea, Suwon 16247, Republic of Korea; onedream1106@naver.com (D.P.); vincentos@naver.com (H.-S.K.); jeong990103@naver.com (S.J.); 2 Department of Statistics and Actuarial Science, Soongsil University, Seoul 06978, Republic of Korea

**Keywords:** osteoporotic fracture, bone mineral density, lifestyle score, physical activity, smoking, alcohol consumption, older women, National Health Insurance Service, cohort study

## Abstract

**Background/Objectives:** Modifiable lifestyle behaviors may be relevant to osteoporotic fracture risk, but their cumulative association across different levels of skeletal health remains unclear. This study investigated the association between a cumulative lifestyle score and osteoporotic fracture risk according to baseline bone mineral density (BMD) category in older women. **Methods:** We conducted a nationwide retrospective cohort study using Korean National Health Insurance Service data from 541,770 women aged 66 years who underwent dual-energy X-ray absorptiometry through the national life-transition health screening program between 2010 and 2016. A cumulative lifestyle score ranging from 0 to 3 was constructed by assigning one point each for regular exercise, non- or ex-smoking status, and non-drinking status. Cox proportional hazards models were used to evaluate associations with any, vertebral, hip, and other osteoporotic fractures across normal BMD, osteopenia, and osteoporosis groups. **Results:** Higher lifestyle scores were generally associated with lower osteoporotic fracture hazards across BMD categories. For any fracture, the adjusted hazard ratios for lifestyle score 3 versus 0 were 0.746 (95% CI, 0.564–0.987) in the normal BMD group, 0.747 (95% CI, 0.630–0.885) in the osteopenia group, and 0.786 (95% CI, 0.668–0.924) in the osteoporosis group. No statistical evidence of interaction between lifestyle score and BMD category was observed. The incidence-rate differences between scores 0 and 3 for any fracture were 4.49, 6.03, and 5.99 per 1000 person-years in the normal BMD, osteopenia, and osteoporosis groups, respectively. **Conclusions:** A more favorable cumulative lifestyle profile was associated with lower osteoporotic fracture risk across BMD categories in older women. Although there was no statistical evidence that the relative associations differed by BMD category, larger incidence-rate differences were observed among women with lower BMD. These observational findings support the potential value of integrated lifestyle assessment as a complementary component of fracture-risk assessment and counseling.

## 1. Introduction

Osteoporotic fractures are a major and growing healthcare burden in aging populations, representing a leading cause of morbidity, functional decline, loss of independence, and long-term care needs in older women, with substantial consequences for quality of life and survival [1,2,3]. As populations age, the burden of fragility fractures is expected to increase further, reinforcing the importance of effective prevention strategies across the later life course [4,5]. Although pharmacologic therapies targeting bone mineral density (BMD) remain central to fracture prevention in women at elevated skeletal risk, medication alone does not fully address the broader clinical context in which fractures occur, and lifestyle assessment may offer a pragmatic, population-oriented complement within preventive healthcare [6,7,8]. Modifiable daily behaviors remain clinically relevant because they are potentially actionable across the population, including among individuals who do not yet meet criteria for pharmacologic treatment [9,10]. Physical activity, smoking, and alcohol consumption have each been associated with bone health and fracture-related outcomes in older adults, suggesting that everyday behavioral patterns represent an important dimension of fracture-risk assessment [11,12,13]. However, examining individual behaviors in isolation may not fully capture the cumulative lifestyle burden relevant to fracture risk [14].

Prior epidemiologic studies have largely evaluated lifestyle factors and fracture risk through a behavior-by-behavior approach, examining smoking, alcohol consumption, or physical activity as separate exposures [13,15]. This body of evidence has provided important insights into the direction and magnitude of individual associations, but such a fragmented framework may not reflect the way health behaviors cluster within individuals in real-world settings [14]. In practice, favorable and unfavorable behaviors rarely occur independently; rather, they tend to co-exist within broader lifestyle patterns that may shape skeletal health and fracture susceptibility over time [14,16]. As a result, the cumulative influence of multiple co-occurring lifestyle behaviors on fracture risk remains incompletely understood, particularly in population-based cohorts of older women.

A simple composite lifestyle score may provide an integrated representation of behavioral exposure across several modifiable domains [16,17]. The rationale for such an approach is not that it offers superior predictive precision or functions as a clinical risk calculator, but that it summarizes the combined behavioral context in which skeletal health is maintained or compromised. Exercise, smoking, and alcohol use may jointly relate to bone strength, fall propensity, physical function, and comorbidity patterns, all of which are relevant to fracture occurrence [17,18,19,20,21]. Framing these exposures cumulatively may therefore offer a clinically interpretable way to examine how everyday lifestyle patterns relate to fracture risk.

The relevance of cumulative lifestyle behaviors may also vary according to baseline skeletal vulnerability. Women with osteopenia or osteoporosis have a substantially higher baseline risk of fracture, and similar relative associations may translate into larger absolute differences in fracture incidence in these groups because of higher underlying risk [8,9]. At the same time, whether any difference across BMD strata reflects true heterogeneity in relative association or primarily differences in baseline risk remains uncertain. Clarifying this distinction is clinically important for interpreting how lifestyle assessment should be framed across levels of underlying skeletal fragility. Therefore, we investigated the association between a simple 3-point cumulative lifestyle score and osteoporotic fracture outcomes, and whether these associations differed according to baseline BMD, in a nationwide cohort of 66-year-old women.

## 2. Methods

### 2.1. Study Design and Data Source

This nationwide retrospective cohort study used data from the Korean National Health Insurance Service (NHIS), a population-based healthcare system covering approximately 97% of the Korean population through a mandatory single-payer system [22,23]. The NHIS database includes linked inpatient and outpatient claims, International Classification of Diseases, 10th Revision (ICD-10) diagnostic codes, prescription records, national health screening results, laboratory and anthropometric measurements, and self-reported lifestyle information obtained through standardized questionnaires [22,23]. Women who participated in the national life-transition health screening program between 1 January 2010 and 31 December 2016 were identified and followed through linked claims data until fracture occurrence, death, or 31 December 2022. The present study evaluated the association between a cumulative lifestyle score and osteoporotic fracture risk across baseline BMD categories.

### 2.2. Study Population

The study population consisted of women aged 66 years who participated in the NHIS-administered national life-transition health screening program and had available baseline DXA-based BMD data. Participants without DXA data were not eligible for the present analysis. The reasons for non-performance of DXA were not available in the archived analytic dataset. For the present analysis, participants were classified as having normal BMD, osteopenia, or osteoporosis according to the DXA-based BMD category recorded in the national health-screening data. Detailed site-specific information regarding the DXA measurement and the precise rule used to derive the recorded BMD category could not be verified in the present analysis.

Participants were excluded if baseline data were missing, if they had a history of vertebral, hip, or other osteoporotic fracture during the 1 year before screening, or if a fracture occurred during the first year after screening. Given the minimal proportion of missing covariate data, complete-case analysis was performed. Follow-up began at the date of the health screening examination and continued until the first qualifying fracture event, death, or 31 December 2022, whichever occurred first. The participant selection process is shown in Supplementary Figure S1.

### 2.3. Exposure Definition

Lifestyle behaviors were assessed at baseline using standardized self-administered questionnaires administered as part of the national health screening program. A cumulative lifestyle score ranging from 0 to 3 was constructed using three prespecified components: (1) regular exercise, defined as vigorous physical activity at least 3 days per week or moderate-intensity physical activity at least 5 days per week; (2) non- or ex-smoking status, including never and former smokers; and (3) non-drinking status, defined as no current alcohol consumption. One point was assigned for each component, and the points were summed to derive the total lifestyle score, with higher scores indicating a more favorable cumulative lifestyle profile.

Equal weighting was selected a priori to maintain simplicity and interpretability and was not intended to imply equivalent biological effects of the individual lifestyle components. The score was developed as a pragmatic cumulative lifestyle measure rather than as a weighted clinical prediction tool.

### 2.4. Outcome Definition

The study outcomes were incident osteoporotic fractures identified from NHIS claims data using ICD-10-based operational definitions consistent with previously validated nationwide Korean fracture studies [24,25]. Fracture outcomes were categorized as any fracture, vertebral fracture, hip fracture, and other osteoporotic fractures. Vertebral fractures were defined by two or more claims with relevant ICD-10 codes (S120, S121, S122, S220, S221, M484, and M485). Hip fractures were identified using ICD-10 codes S720, S721, and S722 in combination with at least one hospitalization record. Other osteoporotic fractures, including fractures of the humerus, wrist, tibia, and fibula, were defined by two or more claims with relevant ICD-10 codes (S420, S422, S423, S525, S526, S823, S825, and S826).

For the analysis of any fracture, only the first qualifying fracture during follow-up was counted. For the hip-fracture analysis, only the first qualifying hip fracture was counted irrespective of laterality; a subsequent contralateral hip fracture was not considered an additional event. Follow-up for each outcome ended at the first qualifying outcome event, death, or 31 December 2022, whichever occurred first.

### 2.5. Covariate Assessment

Covariates were selected a priori based on established epidemiologic evidence regarding fracture risk [8,10]. Sociodemographic variables included income status, derived from health insurance premiums as a proxy for socioeconomic status. Anthropometric variables included body mass index (BMI), categorized as <23.0, 23.0–24.9, and ≥25.0 kg/m^2^, and waist circumference. Clinical measurements included systolic and diastolic blood pressure. Laboratory variables included fasting glucose, total cholesterol, high-density lipoprotein cholesterol, low-density lipoprotein cholesterol, triglycerides, and estimated glomerular filtration rate (eGFR). Diabetes mellitus was identified using ICD-10 diagnostic codes and prescription records. Operational definitions for hypertension, dyslipidemia, chronic kidney disease, and low-income status are provided in Supplementary Table S1.

### 2.6. Statistical Analysis

Baseline characteristics were summarized using means ± standard deviations, geometric means with 95% confidence intervals, or frequencies and percentages, as appropriate. Given the very large sample size, baseline characteristics were interpreted primarily according to the magnitude and direction of differences rather than statistical significance alone. Incidence rates were calculated per 1000 person-years.

Associations between lifestyle score and fracture outcomes were evaluated using Cox proportional hazards regression models, with hazard ratios (HRs) and 95% confidence intervals (CIs). The fully adjusted model included income status, diabetes mellitus, dyslipidemia, hypertension, chronic kidney disease, and BMI and was used as the primary model. The proportional hazards assumption was evaluated by visual inspection of the available log-log survival plot. Kaplan–Meier curves were constructed to illustrate cumulative fracture incidence according to lifestyle score within each BMD category, and differences were evaluated using the log-rank test.

Analyses were performed separately within normal BMD, osteopenia, and osteoporosis groups. Effect modification by BMD category was formally evaluated using multiplicative interaction terms between lifestyle score and BMD category in the fully adjusted Cox models, with *p* values for interaction obtained using Wald tests.

Several sensitivity analyses were performed. First, the Cox models were repeated using lifestyle score 1 rather than score 0 as the reference category. Second, former smokers were excluded, the lifestyle score was recalculated, and the primary analyses were repeated. Third, an additional sensitivity analysis applied a 3-year post-screening exclusion window to assess the potential influence of reverse causation related to early fracture events.

As an exploratory model-comparison analysis, the Akaike information criterion (AIC) and Harrell’s C-index were calculated for models incorporating the composite lifestyle score and models incorporating regular exercise, smoking status, and alcohol consumption as separate variables. Each lifestyle component was also examined separately across BMD categories to evaluate whether the direction of the associations was broadly concordant with that of the cumulative lifestyle score. In addition, all-cause mortality was summarized descriptively as the number of deaths and mortality rate per 1000 person-years.

All statistical analyses were performed using SAS (version 9.4; SAS Institute, Cary, NC, USA) and R software (version 4.4.1; R Foundation, Vienna, Austria). A two-sided *p* value < 0.05 was considered statistically significant.

### 2.7. Ethical Considerations

This study was conducted in accordance with the Declaration of Helsinki and was approved by the Institutional Review Board of the Catholic University of Korea (IRB No. VC25ZISI0084). Because fully anonymized secondary NHIS data were used, the requirement for informed consent was waived in accordance with Korean regulations. Data handling complied with applicable privacy requirements, and NHIS authorization was obtained for data access. Reporting followed the Strengthening the Reporting of Observational Studies in Epidemiology (STROBE) guidelines.

## 3. Results

### 3.1. Cohort Description and Baseline Characteristics

A total of 541,770 women were included, of whom 113,619 (21.0%) had normal BMD, 234,656 (43.3%) had osteopenia, and 193,495 (35.7%) had osteoporosis. Lifestyle scores of 0, 1, 2, and 3 were observed in 0.4%, 6.8%, 72.4%, and 20.4% of participants, respectively. Baseline characteristics varied across BMD categories and lifestyle-score groups. Participants with osteoporosis generally had lower BMI and waist circumference and modest differences in metabolic characteristics compared with those with normal BMD. Higher lifestyle scores were generally accompanied by lower prevalence of hypertension, dyslipidemia, and chronic kidney disease and by more favorable selected metabolic measures (Table 1). Given the large cohort size, these baseline differences were interpreted primarily according to their magnitude and direction rather than statistical significance alone.

### 3.2. Association Between Lifestyle Score and Fracture Outcomes Across BMD Categories

Higher lifestyle scores were generally associated with lower fracture hazards across BMD categories in the fully adjusted models (Table 2). For any fracture, the adjusted HRs for lifestyle score 3 versus 0 were 0.746 (95% CI, 0.564–0.987) in the normal BMD group, 0.747 (95% CI, 0.630–0.885) in the osteopenia group, and 0.786 (95% CI, 0.668–0.924) in the osteoporosis group.

Similar inverse associations were observed for vertebral fracture, with adjusted HRs for score 3 versus 0 of 0.437 (95% CI, 0.261–0.732), 0.691 (95% CI, 0.494–0.967), and 0.656 (95% CI, 0.503–0.856) across the normal BMD, osteopenia, and osteoporosis groups, respectively. For hip fracture, the corresponding HRs were 0.613 (95% CI, 0.252–1.491), 0.459 (95% CI, 0.290–0.726), and 0.350 (95% CI, 0.240–0.509). Associations for other fractures were weaker and less consistent across BMD categories.

### 3.3. Incidence-Rate Differences According to BMD Category

**Figure 1:**
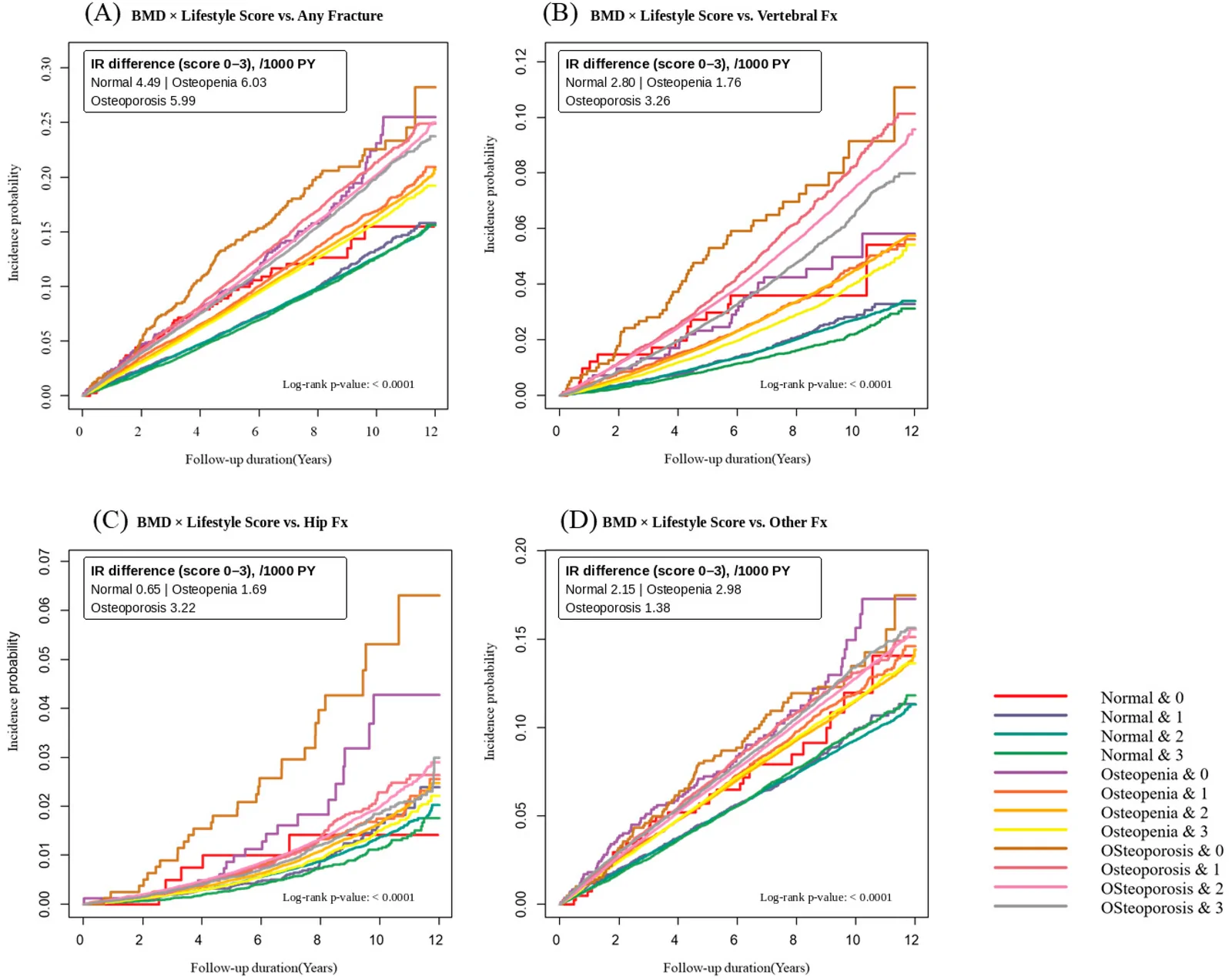
Kaplan–Meier cumulative incidence curves for osteoporotic fractures according to baseline bone mineral density category and lifestyle score. Cumulative incidence of (**A**) any fracture, (**B**) vertebral fracture, (**C**) hip fracture, and (**D**) other fractures according to baseline BMD category (normal, osteopenia, osteoporosis) and lifestyle score (0–3). Higher scores represent more favorable lifestyle profiles. Numeric annotations indicate the incidence-rate difference between lifestyle scores 0 and 3 (score 0 minus score 3), expressed per 1000 person-years within each BMD category. Log-rank *p* < 0.0001 for all outcomes. BMD, bone mineral density.

Although the relative associations were broadly similar across BMD categories, the incidence-rate differences between lifestyle scores 0 and 3 tended to be larger among women with lower BMD. For any fracture, the incidence-rate difference was 4.49 per 1000 person-years in the normal BMD group, 6.03 in the osteopenia group, and 5.99 in the osteoporosis group. In the osteoporosis group, the corresponding differences were 3.26 per 1000 person-years for vertebral fracture and 3.22 per 1000 person-years for hip fracture. Numeric annotations in Figure 1 show the incidence-rate difference between lifestyle scores 0 and 3 within each BMD category.

Kaplan–Meier curves demonstrated separation according to lifestyle score within BMD categories (Figure 1). Visual inspection of the available log-log survival plot showed approximately parallel trajectories and no clear indication of a major violation in the displayed comparison (Supplementary Figure S2).

### 3.4. Interaction and Sensitivity Analyses

No statistically significant interaction was detected between lifestyle score and BMD category for any fracture (*p* for interaction = 0.954), vertebral fracture (*p* = 0.389), hip fracture (*p* = 0.714), or other fracture (*p* = 0.942). These findings indicate an absence of statistical evidence for heterogeneity across BMD categories rather than equivalence of the associations between strata.

Using lifestyle score 1 as the reference category produced broadly similar directional patterns, although several estimates were attenuated (Supplementary Table S3). Excluding former smokers and recalculating the lifestyle score did not materially alter the primary findings. For any fracture, the adjusted HRs for score 3 versus 0 were 0.743 (95% CI, 0.562–0.984), 0.745 (95% CI, 0.629–0.883), and 0.783 (95% CI, 0.665–0.921) in the normal BMD, osteopenia, and osteoporosis groups, respectively (Supplementary Table S5).

In the 3-year lag sensitivity analysis, the associations were generally maintained but attenuated in some strata. For any fracture, the adjusted HRs for score 3 versus 0 were 0.905 (95% CI, 0.638–1.283), 0.781 (95% CI, 0.640–0.955), and 0.793 (95% CI, 0.656–0.959) in the normal BMD, osteopenia, and osteoporosis groups, respectively (*p* for interaction = 0.546; Supplementary Table S4).

### 3.5. Individual Lifestyle Components and Exploratory Model Comparison

In the component-level analyses, smoking, alcohol consumption, and regular exercise showed heterogeneous patterns of association across fracture outcomes (Table 3; Supplementary Table S2). Current smoking was associated with higher fracture hazards for several outcomes, whereas regular exercise showed inverse associations particularly for vertebral and hip fractures. Associations with current alcohol consumption were less consistent across outcomes and BMD categories. No statistically significant interaction between individual lifestyle components and BMD category was observed.

In an exploratory model-comparison analysis, the model incorporating the composite lifestyle score had a lower AIC (1,843,758.6 vs. 1,846,129.7) and a higher Harrell’s C-index (0.5563 vs. 0.5211) than the model incorporating exercise, smoking, and alcohol consumption as separate variables (Supplementary Table S7). The overall discriminatory ability remained modest.

### 3.6. Mortality During Follow-Up

During follow-up, 22,160 deaths occurred over 4,264,133.12 person-years, corresponding to an overall mortality rate of 5.20 per 1000 person-years. Mortality rates were 5.03, 4.94, and 5.61 per 1000 person-years in the normal BMD, osteopenia, and osteoporosis groups, respectively. Across lifestyle scores 0, 1, 2, and 3, the corresponding mortality rates were 10.25, 5.60, 5.36, and 4.39 per 1000 person-years, respectively (Supplementary Table S6).

## 4. Discussion

In this nationwide cohort of 66-year-old women, a more favorable cumulative lifestyle profile was associated with lower osteoporotic fracture hazards across baseline BMD categories. No statistically significant interaction was detected between lifestyle score and BMD category, indicating no formal evidence that the relative associations differed according to skeletal status. However, similar relative associations occurred against substantially different baseline fracture rates, resulting in larger incidence-rate differences among women with osteopenia or osteoporosis. These findings highlight the importance of considering both relative and absolute measures when interpreting lifestyle-related associations across levels of skeletal vulnerability.

The present findings are broadly aligned with previous epidemiologic evidence linking individual lifestyle behaviors with fracture outcomes. Meta-analyses have reported associations of exercise, smoking, and alcohol consumption with fracture risk, while a Korean nationwide cohort demonstrated lower fracture risk following smoking cessation compared with continued smoking [11,12,13,15]. Beyond individual behaviors, previous work has also suggested that greater adherence to multiple favorable lifestyle factors is associated with lower osteoporosis and fracture risk across levels of genetic susceptibility [16]. Our study extends this literature by examining a simple cumulative lifestyle profile within BMD-defined strata in an age-homogeneous population of older women. Differences in the magnitude of associations across studies may reflect differences in population characteristics, definitions of lifestyle behaviors, fracture ascertainment, and underlying skeletal risk.

Health behaviors commonly cluster within individuals rather than occurring in isolation, and cumulative assessment may therefore provide a pragmatic representation of an individual’s overall behavioral profile [14,16,17]. In the present study, the individual components showed less uniform patterns across fracture outcomes than the cumulative score. Nevertheless, the composite score should not be interpreted as demonstrating that the three behaviors exert equivalent biological effects or as establishing superiority over individual behavioral assessment. In the exploratory model comparison, the composite score showed modestly better model fit and discrimination than the representation of the three lifestyle components separately; however, the overall C-index remained low, indicating limited discriminatory ability and precluding interpretation of the score as a clinical prediction tool.

Several biological pathways may underlie the observed associations. Smoking has been linked to impaired bone health through oxidative stress, altered estrogen metabolism, and an imbalance between bone formation and resorption [19,26]. Excessive alcohol consumption may adversely affect osteoblast function and calcium and vitamin D homeostasis, although fracture associations appear to depend on the amount and pattern of consumption [13,20,27]. Regular physical activity may contribute to bone strength and also influence muscle function and balance through mechanical loading and muscle-bone interactions [11,18,28,29]. These pathways may co-exist within individuals and provide biological plausibility for examining lifestyle behaviors cumulatively. However, the observational design of the present study does not establish that modification of these behaviors would causally reduce fracture risk.

The smoking and alcohol components warrant particularly cautious interpretation. Former smokers were classified together with never smokers in the primary score because both represented absence of current smoking; however, prior smoking exposure may remain relevant to fracture risk. Reassuringly, exclusion of former smokers and recalculation of the lifestyle score did not materially alter the primary findings. For alcohol, current non-drinkers could not be separated into lifelong abstainers and individuals who had stopped drinking because of illness, frailty, medication use, or functional decline. Accordingly, inverse associations involving non-drinking should not be interpreted as evidence of a protective or harmful effect of alcohol, and reverse causation and sick-quitter bias remain possible.

From a preventive healthcare perspective, the score may offer a simple framework for organizing lifestyle assessment and counseling using information already collected during routine health screening. Its potential relevance may be greatest for risk communication in women with osteopenia or osteoporosis, not because the relative associations were demonstrably stronger in these groups, but because their higher underlying fracture rates translated similar relative associations into larger incidence-rate differences. Such lifestyle assessment should complement, rather than replace, BMD-based risk assessment and established pharmacologic management [8,30].

This study has several strengths. It used a large nationwide cohort with an age-homogeneous population of 66-year-old women, reducing confounding by age and allowing comparisons across participants with a similar baseline life stage. BMD was assessed using DXA-based screening data, fracture outcomes were ascertained longitudinally using claims-based operational definitions, and the relatively long follow-up period enabled evaluation of multiple osteoporotic fracture outcomes. In addition, the analysis considered both relative associations and incidence-rate differences across BMD strata and incorporated several sensitivity analyses addressing reference-group selection, former smoking, and potential reverse causation.

Several limitations should be considered. First, this was an observational study, and causal inference cannot be made. Residual confounding remains possible despite multivariable adjustment. Important fracture-related factors—including fall history, frailty, sarcopenia, dietary calcium and vitamin D intake or supplementation, osteoporosis medication, systemic corticosteroid use, hormone replacement therapy, and nutritional status—were not available or incorporated in the analysis.

Second, lifestyle behaviors were self-reported and assessed only at baseline. Misclassification is therefore possible, and changes in exercise, smoking, or alcohol consumption during follow-up could not be captured. Reverse causation is also possible because underlying frailty or subclinical functional decline may influence both lifestyle behaviors and subsequent fracture risk. Although associations were generally maintained in the 3-year lag sensitivity analysis, attenuation in some strata indicates that this possibility cannot be excluded.

Third, the simplified lifestyle score assigned equal weight to exercise, smoking status, and alcohol consumption and did not account for differences in dose, duration, frequency, or intensity. Former smokers were grouped with never smokers in the primary score, and current non-drinkers could not be distinguished from former drinkers or lifelong abstainers. These simplified classifications improve practicality but may introduce exposure misclassification and should be considered when interpreting the component-specific findings.

Fourth, the lifestyle score 0 group was small, both overall and within individual BMD strata. This may have reduced the precision of estimates using score 0 as the reference and limited statistical power to detect interaction by BMD category. Sensitivity analyses using lifestyle score 1 as the reference showed broadly similar directional patterns, but the non-significant interaction tests should be interpreted as an absence of statistical evidence for heterogeneity rather than evidence of equivalent associations across BMD strata.

Fifth, a substantial number of otherwise eligible women did not have available DXA measurements and were excluded. Because the reasons for DXA non-performance and the characteristics of excluded participants could not be evaluated in the archived analytic dataset, selection bias related to DXA availability cannot be excluded. Detailed site-specific information regarding DXA measurement and the precise rule used to derive the recorded BMD category also could not be verified in the present analysis. In addition, the study population consisted exclusively of 66-year-old Korean women participating in a national health-screening program, which may limit generalizability to men, other age groups, and other populations.

## 5. Conclusions

A more favorable cumulative lifestyle profile was associated with lower osteoporotic fracture risk across BMD categories in this nationwide cohort of older women. Although there was no statistical evidence that the relative associations differed by BMD category, larger incidence-rate differences were observed among women with osteopenia or osteoporosis because of their higher underlying fracture rates. These observational findings support the potential value of integrated lifestyle assessment as a complementary component of fracture-risk assessment and counseling, without implying that lifestyle modification causally reduces fracture risk or replaces established BMD-based and pharmacologic approaches.

## Figures and Tables

**Table 1:** Baseline characteristics of the study population by bone mineral density category and cumulative lifestyle score.

Characteristic	BMD Category			*p* Value	Cumulative Lifestyle Score				*p* Value
	Normal	Osteopenia	Osteoporosis		Score 0	Score 1	Score 2	Score 3	
n	113,619	234,656	193,495		2019	37,044	392,211	110,496	
Low-income status *	24,078 (21.19)	50,908 (21.69)	45,378 (23.45)	<0.0001	845 (41.85)	10,638 (28.72)	88,126 (22.47)	20,755 (18.78)	<0.0001
**Smoking status**				**<0.0001**					**<0.0001**
Never smoker	110,475 (97.23)	228,254 (97.27)	187,579 (96.94)		0 (0)	29,550 (79.77)	387,205 (98.72)	109,553 (99.15)	
Former smoker	1380 (1.21)	2578 (1.10)	1887 (0.98)		0 (0)	1088 (2.94)	3814 (0.97)	943 (0.85)	
Current smoker	1764 (1.55)	3824 (1.63)	4029 (2.08)		2019 (100)	6406 (17.29)	1192 (0.30)	0 (0)	
**Alcohol consumption**				**<0.0001**					**<0.0001**
None	103,359 (90.97)	215,300 (91.75)	178,958 (92.49)		0 (0)	5979 (16.14)	381,142 (97.18)	110,496 (100)	
Mild	9913 (8.72)	18,827 (8.02)	14,107 (7.29)		1825 (90.39)	30,200 (81.52)	10,822 (2.76)	0 (0)	
Heavy	347 (0.31)	529 (0.23)	430 (0.22)		194 (9.61)	865 (2.34)	247 (0.06)	0 (0)	
Regular exercise	28,003 (24.65)	54,842 (23.37)	40,339 (20.85)	<0.0001	0 (0)	427 (1.15)	12,261 (3.13)	110,496 (100)	<0.0001
Diabetes mellitus	25,704 (22.62)	42,560 (18.14)	29,072 (15.02)	<0.0001	366 (18.13)	6276 (16.94)	70,982 (18.10)	19,712 (17.84)	<0.0001
Hypertension	67,890 (59.75)	129,225 (55.07)	98,325 (50.82)	<0.0001	1131 (56.02)	20,709 (55.90)	214,788 (54.76)	58,812 (53.23)	<0.0001
Dyslipidemia	60,233 (53.01)	117,407 (50.03)	88,958 (45.97)	<0.0001	1004 (49.73)	18,569 (50.13)	191,780 (48.90)	55,245 (50.00)	<0.0001
Chronic kidney disease	14,968 (13.17)	26,352 (11.23)	19,018 (9.83)	<0.0001	233 (11.54)	3643 (9.83)	44,163 (11.26)	12,299 (11.13)	<0.0001
Height, cm	154.67 ± 5.05	153.85 ± 5.05	152.74 ± 5.18	<0.0001	153.60 ± 5.01	153.46 ± 5.13	153.48 ± 5.17	154.19 ± 5.03	<0.0001
Weight, kg	60.86 ± 8.30	58.38 ± 7.77	55.45 ± 7.73	<0.0001	56.50 ± 8.69	58.22 ± 8.29	57.85 ± 8.20	57.76 ± 7.78	<0.0001
Body mass index, kg/m^2^	25.43 ± 3.25	24.66 ± 3.07	23.76 ± 3.08	<0.0001	23.93 ± 3.40	24.71 ± 3.24	24.55 ± 3.21	24.29 ± 3.02	<0.0001
Waist circumference, cm	82.97 ± 8.33	81.37 ± 8.06	79.51 ± 8.12	<0.0001	80.92 ± 8.74	81.87 ± 8.43	81.20 ± 8.29	80.23 ± 7.90	<0.0001
Systolic blood pressure, mmHg	128.00 ± 14.88	127.35 ± 14.97	126.87 ± 15.25	<0.0001	124.87 ± 16.02	127.19 ± 15.30	127.43 ± 15.12	127.00 ± 14.71	<0.0001
Diastolic blood pressure, mmHg	76.41 ± 9.45	76.40 ± 9.50	76.37 ± 9.61	0.4649	75.31 ± 9.92	76.79 ± 9.73	76.42 ± 9.54	76.15 ± 9.38	<0.0001
High-density lipoprotein cholesterol, mg/dL	54.84 ± 15.27	55.49 ± 15.30	56.14 ± 15.88	<0.0001	58.50 ± 15.21	57.85 ± 16.73	55.16 ± 15.37	56.27 ± 15.48	<0.0001
Low-density lipoprotein cholesterol, mg/dL	117.99 ± 36.93	119.84 ± 36.40	121.02 ± 36.20	<0.0001	117.34 ± 37.52	121.58 ± 36.60	119.88 ± 36.55	119.31 ± 36.02	<0.0001
Fasting glucose, mg/dL	104.80 ± 26.32	102.54 ± 23.69	100.82 ± 22.62	<0.0001	104.21 ± 25.44	103.16 ± 24.30	102.44 ± 24.22	101.97 ± 22.74	<0.0001
Total cholesterol, mg/dL	198.51 ± 39.75	200.70 ± 39.15	202.12 ± 39.02	<0.0001	204.20 ± 39.45	205.05 ± 39.68	200.56 ± 39.32	199.93 ± 38.77	<0.0001
Estimated glomerular filtration rate, mL/min/1.73 m^2^	79.06 ± 15.52	80.28 ± 15.10	81.60 ± 14.98	<0.0001	80.83 ± 15.35	81.34 ± 14.68	80.48 ± 15.27	80.27 ± 15.00	<0.0001
Triglycerides †, mg/dL	113.53 (113.21, 113.86)	112.32 (112.09, 112.54)	110.49 (110.25, 110.74)	<0.0001	125.98 (123.32, 128.70)	113.11 (112.54, 113.69)	113.01 (112.84, 113.18)	107.49 (107.18, 107.80)	<0.0001

Values are presented as n (%), mean ± standard deviation, or geometric mean (95% confidence interval), as appropriate. * Low-income status was defined as the lowest income quartile and/or Medical Aid beneficiary status. † Triglyceride levels are presented as geometric mean (95% confidence interval). Abbreviation: BMD, bone mineral density.

**Table 2:** Association between cumulative lifestyle score and fracture outcomes according to bone mineral density category.

Fracture Outcome	Bone Mineral Density Category	Lifestyle Score	Participants, n	Fracture Events, n	Follow-Up Duration, Person-Years	Incidence Rate per 1000 Person-Years	Adjusted Hazard Ratio (95% CI)
**Any fracture**	**Normal bone mineral density**	**0**	**407**	**50**	**2886.30**	**17.32**	**1.00 (reference)**
		1	8066	795	59,210.91	13.43	0.779 (0.585, 1.036)
		2	80,287	7858	598,461.10	13.13	0.758 (0.574, 1.001)
		3	24,859	2375	185,123.23	12.83	0.746 (0.564, 0.987)
	Osteopenia	0	824	136	5908.82	23.02	1.00 (reference)
		1	15,878	2102	114,729.33	18.32	0.804 (0.676, 0.956)
		2	168,766	21,764	1,241,606.44	17.53	0.767 (0.648, 0.907)
		3	49,188	6120	360,303.78	16.99	0.747 (0.630, 0.885)
	Osteoporosis	0	788	149	5485.20	27.16	1.00 (reference)
		1	13,100	2175	93,246.62	23.33	0.865 (0.733, 1.021)
		2	143,158	22,616	1,034,695.94	21.86	0.809 (0.689, 0.950)
		3	36,449	5569	263,064.85	21.17	0.786 (0.668, 0.924)
	***p* for interaction**						**0.9543**
**Vertebral fracture**	**Normal bone mineral density**	**0**	**407**	**15**	**3045.29**	**4.93**	**1.00 (reference)**
		1	8066	164	61,758.02	2.66	0.532 (0.313, 0.902)
		2	80,287	1607	624,345.48	2.57	0.516 (0.310, 0.858)
		3	24,859	412	193,072.37	2.13	0.437 (0.261, 0.732)
	Osteopenia	0	824	35	6308.04	5.55	1.00 (reference)
		1	15,878	528	121,334.65	4.35	0.776 (0.551, 1.092)
		2	168,766	5706	1,310,950.11	4.35	0.779 (0.558, 1.086)
		3	49,188	1439	380,114.35	3.79	0.691 (0.494, 0.967)
	Osteoporosis	0	788	56	5895.44	9.50	1.00 (reference)
		1	13,100	804	99,242.56	8.10	0.837 (0.638, 1.097)
		2	143,158	7997	1,098,261.01	7.28	0.758 (0.583, 0.986)
		3	36,449	1746	279,963.55	6.24	0.656 (0.503, 0.856)
	***p* for interaction**						**0.3888**
**Hip fracture**	**Normal bone mineral density**	**0**	**407**	**5**	**3100.41**	**1.61**	**1.00 (reference)**
		1	8066	77	62,191.33	1.24	0.793 (0.321, 1.961)
		2	80,287	749	627,990.59	1.19	0.744 (0.309, 1.792)
		3	24,859	187	194,063.32	0.96	0.613 (0.252, 1.491)
	Osteopenia	0	824	19	6396.25	2.97	1.00 (reference)
		1	15,878	199	122,724.82	1.62	0.587 (0.367, 0.940)
		2	168,766	1931	1,326,762.27	1.46	0.515 (0.327, 0.809)
		3	49,188	491	383,940.68	1.28	0.459 (0.290, 0.726)
	Osteoporosis	0	788	29	6041.32	4.80	1.00 (reference)
		1	13,100	185	101,773.71	1.82	0.405 (0.274, 0.599)
		2	143,158	2023	1,123,956.84	1.80	0.396 (0.274, 0.571)
		3	36,449	451	285,291.45	1.58	0.350 (0.240, 0.509)
	***p* for interaction**						**0.7140**
**Other fractures**	**Normal bone mineral density**	**0**	**407**	**36**	**2962.94**	**12.15**	**1.00 (reference)**
		1	8066	594	59,928.00	9.91	0.822 (0.587, 1.151)
		2	80,287	5882	605,691.53	9.71	0.802 (0.578, 1.113)
		3	24,859	1870	186,979.98	10.00	0.826 (0.594, 1.148)
	Osteopenia	0	824	93	6035.82	15.41	1.00 (reference)
		1	15,878	1502	117,092.35	12.83	0.843 (0.684, 1.040)
		2	168,766	15,491	1,265,614.82	12.24	0.802 (0.654, 0.983)
		3	49,188	4549	366,094.59	12.43	0.813 (0.662, 0.998)
	Osteoporosis	0	788	89	5744.72	15.49	1.00 (reference)
		1	13,100	1372	96,411.49	14.23	0.933 (0.753, 1.156)
		2	143,158	14,528	1,067,670.05	13.61	0.889 (0.722, 1.095)
		3	36,449	3808	269,892.40	14.11	0.921 (0.746, 1.136)
	***p* for interaction**						**0.9423**

Data are shown as number of participants, number of fracture events, follow-up duration in person-years, incidence rate per 1000 person-years, and adjusted hazard ratio (95% confidence interval). Adjusted hazard ratios were estimated using Cox proportional hazards regression models and adjusted for income status, diabetes mellitus, dyslipidemia, hypertension, chronic kidney disease, and body mass index. Within each BMD category, lifestyle score 0 served as the reference group. *p* for interaction was calculated to evaluate whether the association between lifestyle score and fracture risk differed by BMD category. Abbreviation: CI, confidence interval.

**Table 3:** Adjusted associations of individual lifestyle components with fracture outcomes by bone mineral density category.

Lifestyle Factor	Fracture Outcome	Comparison	Normal BMD	Osteopenia	Osteoporosis	*p* for Interaction
Smoking status	Any fracture	Former smoker	1.035 (0.874, 1.225)	1.121 (1.012, 1.243)	1.180 (1.060, 1.313)	0.1779
Smoking status	Any fracture	Current smoker	1.087 (0.942, 1.256)	1.126 (1.035, 1.225)	1.246 (1.160, 1.338)	
Smoking status	Vertebral fracture	Former smoker	1.134 (0.786, 1.636)	1.152 (0.942, 1.410)	1.264 (1.060, 1.508)	0.9138
Smoking status	Vertebral fracture	Current smoker	1.543 (1.171, 2.032)	1.401 (1.204, 1.630)	1.455 (1.299, 1.630)	
Smoking status	Hip fracture	Former smoker	1.432 (0.886, 2.314)	1.631 (1.221, 2.178)	1.543 (1.124, 2.118)	0.5961
Smoking status	Hip fracture	Current smoker	1.501 (1.009, 2.234)	1.956 (1.580, 2.422)	2.139 (1.783, 2.567)	
Smoking status	Other fractures	Former smoker	1.017 (0.838, 1.236)	1.091 (0.965, 1.233)	1.098 (0.958, 1.259)	0.5728
Smoking status	Other fractures	Current smoker	0.966 (0.811, 1.150)	0.952 (0.856, 1.060)	1.061 (0.966, 1.166)	
**Alcohol consumption**	**Any fracture**	**Yes**	**1.051 (0.986, 1.121)**	**1.055 (1.013, 1.099)**	**1.014 (0.972, 1.059)**	**0.3878**
Alcohol consumption	Vertebral fracture	Yes	1.030 (0.891, 1.191)	0.927 (0.852, 1.008)	1.000 (0.930, 1.075)	0.2926
Alcohol consumption	Hip fracture	Yes	0.888 (0.706, 1.116)	0.949 (0.822, 1.095)	0.837 (0.717, 0.978)	0.5059
Alcohol consumption	Other fractures	Yes	1.085 (1.008, 1.167)	1.104 (1.054, 1.158)	1.041 (0.988, 1.097)	0.2549
**Regular exercise**	**Any fracture**	**Yes**	**0.994 (0.952, 1.038)**	**0.980 (0.954, 1.007)**	**0.970 (0.943, 0.997)**	**0.6371**
Regular exercise	Vertebral fracture	Yes	0.862 (0.779, 0.955)	0.891 (0.843, 0.941)	0.867 (0.826, 0.911)	0.7378
Regular exercise	Hip fracture	Yes	0.802 (0.688, 0.934)	0.882 (0.803, 0.970)	0.890 (0.808, 0.981)	0.4983
Regular exercise	Other fractures	Yes	1.044 (0.994, 1.097)	1.022 (0.991, 1.055)	1.034 (1.000, 1.070)	0.7441

Values are adjusted hazard ratios (95% confidence intervals). This abbreviated main table presents effect estimates only; full participant counts, event counts, person-years, and incidence rates are provided in Supplementary Table S2. For smoking status, never smokers served as the reference group. For alcohol consumption and regular exercise, the “No” group served as the reference group. Adjusted hazard ratios were estimated using Cox proportional hazards regression models and adjusted for income status, diabetes mellitus, dyslipidemia, hypertension, chronic kidney disease, and body mass index. *p* for interaction was calculated to evaluate whether the association between each lifestyle component and fracture risk differed by BMD category. Abbreviation: BMD, bone mineral density.

## Data Availability

Restrictions apply to the availability of these data. The data were obtained from the Korean National Health Insurance Service (NHIS) and are available from the NHIS upon reasonable request and with permission from the NHIS. The authors do not have permission to redistribute the data.

## Supplementary Materials

The following supporting information can be downloaded at: https://www.mdpi.com/article/10.3390/healthcare14172812/s1. Table S1: Operational definitions of selected comorbidities and socioeconomic status; Table S2: Detailed associations of smoking status, alcohol consumption, and regular exercise with fracture outcomes according to bone mineral density category; Table S3: Sensitivity analysis of the association between lifestyle score and fracture outcomes using lifestyle score 1 as the reference category; Table S4: Sensitivity analysis of the association between lifestyle score and fracture outcomes using a 3-year lag period; Table S5: Sensitivity analysis after excluding former smokers; Table S6: All-cause mortality rates according to bone mineral density category and lifestyle score; Table S7: Exploratory comparison of model fit and discrimination between the composite lifestyle score and individual lifestyle components; Figure S1: Flowchart illustrating the selection process for the study population; Figure S2: Available log-log survival plot for visual assessment of the proportional hazards assumption.
