# Supplementary material for: Cumulative Lifestyle Profiles and Osteoporotic Fracture Risk in Older Women: A Nationwide Korean Cohort Study
Source: Healthcare (Basel). 2026 Sep 2;14(17):2812. doi: 10.3390/healthcare14172812 (PMC13565032; doi:10.3390/healthcare14172812)
Supplement: Supplementary file 1 [file healthcare-14-02812-s001.zip › healthcare-4469441-supplementary.pdf]

Supplementary Table S1. Operational definitions of selected comorbidities and socioeconomic status

	ICD-10 codes	Additional definitions
**Past medical history**		
Hypertension[a]	I10-I13, I15	Claims for antihypertensive agents<br>or SBP ≥ 140mmHg or DBP ≥ 90mmHg
Dyslipidemia	E78	Claims for agents for dyslipidemia<br>or Total cholesterol ≥ 240 mg/dL
Chronic kidney disease		Estimated glomerular filtration rate (eGFR) of < 60 mL/min/1.73 m^2
**Socioeconomic status**		
Low income		Composite of the lowest quartile of yearly income in addition to medicare beneficiaries

[a]Hypertension was identified to have diseases when patients had either one diagnosis during hospitalization or twice or more diagnoses at outpatient clinics for preventing overestimation of diagnosis.

Supplementary Table S2. Detailed associations of smoking status, alcohol consumption, and regular exercise with fracture outcomes according to bone mineral density category

Lifestyle factor	Fracture outcome	Bone mineral density category	Category	Participants, n	Fracture events, n	Follow-up duration, person-years	Incidence rate per 1,000 person-years	Adjusted hazard ratio (95% CI)
**Smoking status**	**Any fracture**	**Normal bone mineral density**	**Never smoker**	**110475**	**10752**	**822850.77**	**13.07**	**1.00 (reference)**
			Former smoker	1380	137	9965.59	13.75	1.035 (0.874, 1.225)
			Current smoker	1764	189	12865.17	14.69	1.087 (0.942, 1.256)
		Osteopenia	Never smoker	228254	29197	1676541.32	17.42	1.00 (reference)
			Former smoker	2578	368	18463.53	19.93	1.121 (1.012, 1.243)
			Current smoker	3824	557	27543.52	20.22	1.126 (1.035, 1.225)
		Osteoporosis	Never smoker	187579	29388	1355213.49	21.69	1.00 (reference)
			Former smoker	1887	339	13044.39	25.99	1.180 (1.060, 1.313)
			Current smoker	4029	782	28234.73	27.70	1.246 (1.160, 1.338)
		**P for interaction**						**0.1779**
	**Vertebral fracture**	**Normal bone mineral density**	**Never smoker**	**110475**	**2117**	**858439.76**	**2.47**	**1.00 (reference)**
			Former smoker	1380	29	10397.03	2.79	1.134 (0.786, 1.636)
			Current smoker	1764	52	13384.37	3.89	1.543 (1.171, 2.032)
		Osteopenia	Never smoker	228254	7439	1769967.52	4.20	1.00 (reference)
			Former smoker	2578	96	19619.16	4.89	1.152 (0.942, 1.410)
			Current smoker	3824	173	29120.46	5.94	1.401 (1.204, 1.630)
		Osteoporosis	Never smoker	187579	10165	1439027.41	7.06	1.00 (reference)
			Former smoker	1887	126	14034.81	8.98	1.264 (1.060, 1.508)
			Current smoker	4029	312	30300.36	10.30	1.455 (1.299, 1.630)
		**P for interaction**						**0.9138**
	**Hip fracture**	**Normal bone mineral density**	**Never smoker**	**110475**	**976**	**863367.10**	**1.13**	**1.00 (reference)**

Lifestyle factor	Fracture outcome	Bone mineral density category	Category	Participants, n	Fracture events, n	Follow-up duration, person-years	Incidence rate per 1,000 person-years	Adjusted hazard ratio (95% CI)
			Former smoker	1380	17	10430.14	1.63	1.432 (0.886, 2.314)
			Current smoker	1764	25	13548.41	1.85	1.501 (1.009, 2.234)
		Osteopenia	Never smoker	228254	2505	1790466.75	1.40	1.00 (reference)
			Former smoker	2578	47	19842.82	2.37	1.631 (1.221, 2.178)
			Current smoker	3824	88	29514.46	2.98	1.956 (1.580, 2.422)
		Osteoporosis	Never smoker	187579	2526	1471541.81	1.72	1.00 (reference)
			Former smoker	1887	39	14384.42	2.71	1.543 (1.124, 2.118)
			Current smoker	4029	123	31137.10	3.95	2.139 (1.783, 2.567)
		**P for interaction**						**0.5961**
	**Other fractures**	**Normal bone mineral density**	**Never smoker**	**110475**	**8151**	**832386.60**	**9.79**	**1.00 (reference)**
			Former smoker	1380	103	10075.45	10.22	1.017 (0.838, 1.236)
			Current smoker	1764	128	13100.39	9.77	0.966 (0.811, 1.150)
		Osteopenia	Never smoker	228254	21031	1707637.95	12.32	1.00 (reference)
			Former smoker	2578	260	18878.44	13.77	1.091 (0.965, 1.233)
			Current smoker	3824	344	28321.19	12.15	0.952 (0.856, 1.060)
		Osteoporosis	Never smoker	187579	19144	1396536.14	13.71	1.00 (reference)
			Former smoker	1887	209	13597.61	15.37	1.098 (0.958, 1.259)
			Current smoker	4029	444	29584.90	15.01	1.061 (0.966, 1.166)
		**P for interaction**						**0.5728**
**Alcohol consumption**	**Any fracture**	**Normal bone mineral density**	**No**	**103359**	**10054**	**770808.23**	**13.04**	**1.00 (reference)**
			Yes	10260	1024	74873.30	13.68	1.051 (0.986, 1.121)
		Osteopenia	No	215300	27548	1582631.64	17.41	1.00 (reference)

Lifestyle factor	Fracture outcome	Bone mineral density category	Category	Participants, n	Fracture events, n	Follow-up duration, person-years	Incidence rate per 1,000 person-years	Adjusted hazard ratio (95% CI)
			Yes	19356	2574	139916.74	18.40	1.055 (1.013, 1.099)
		Osteoporosis	No	178958	28205	1292904.41	21.82	1.00 (reference)
			Yes	14537	2304	103588.20	22.24	1.014 (0.972, 1.059)
		**P for interaction**						**0.3878**
	**Vertebral fracture**	**Normal bone mineral density**	**No**	**103359**	**1996**	**804001.79**	**2.48**	**1.00 (reference)**
			Yes	10260	202	78219.37	2.58	1.030 (0.891, 1.191)
		Osteopenia	No	215300	7111	1670472.68	4.26	1.00 (reference)
			Yes	19356	597	148234.46	4.03	0.927 (0.852, 1.008)
		Osteoporosis	No	178958	9795	1373258.11	7.13	1.00 (reference)
			Yes	14537	808	110104.46	7.34	1.000 (0.930, 1.075)
		**P for interaction**						**0.2926**
	**Hip fracture**	**Normal bone mineral density**	**No**	**103359**	**938**	**808575.07**	**1.16**	**1.00 (reference)**
			Yes	10260	80	78770.58	1.02	0.888 (0.706, 1.116)
		Osteopenia	No	215300	2435	1690025.54	1.44	1.00 (reference)
			Yes	19356	205	149798.49	1.37	0.949 (0.822, 1.095)
		Osteoporosis	No	178958	2516	1404387.06	1.79	1.00 (reference)
			Yes	14537	172	112676.26	1.53	0.837 (0.717, 0.978)
		**P for interaction**						**0.5059**
	**Other fractures**	**Normal bone mineral density**	**No**	**103359**	**7587**	**779834.10**	**9.73**	**1.00 (reference)**
			Yes	10260	795	75728.35	10.50	1.085 (1.008, 1.167)
		Osteopenia	No	215300	19721	1612445.99	12.23	1.00 (reference)
			Yes	19356	1914	142391.58	13.44	1.104 (1.054, 1.158)

Lifestyle factor	Fracture outcome	Bone mineral density category	Category	Participants, n	Fracture events, n	Follow-up duration, person-years	Incidence rate per 1,000 person-years	Adjusted hazard ratio (95% CI)
		Osteoporosis	No	178958	18281	1333012.78	13.71	1.00 (reference)
			Yes	14537	1516	106705.87	14.21	1.041 (0.988, 1.097)
		**P for interaction**						**0.2549**
**Regular exercise**	**Any fracture**	**Normal bone mineral density**	**No**	**85616**	**8385**	**637803.33**	**13.15**	**1.00 (reference)**
			Yes	28003	2693	207878.20	12.95	0.994 (0.952, 1.038)
		Osteopenia	No	179814	23245	1321331.31	17.59	1.00 (reference)
			Yes	54842	6877	401217.07	17.14	0.980 (0.954, 1.007)
		Osteoporosis	No	153156	24327	1105821.86	22.00	1.00 (reference)
			Yes	40339	6182	290670.75	21.27	0.970 (0.943, 0.997)
		**P for interaction**						**0.6371**
	**Vertebral fracture**	**Normal bone mineral density**	**No**	**85616**	**1726**	**665393.66**	**2.59**	**1.00 (reference)**
			Yes	28003	472	216827.50	2.18	0.862 (0.779, 0.955)
		Osteopenia	No	179814	6097	1395188.61	4.37	1.00 (reference)
			Yes	54842	1611	423518.54	3.80	0.891 (0.843, 0.941)
		Osteoporosis	No	153156	8653	1174027.64	7.37	1.00 (reference)
			Yes	40339	1950	309334.93	6.30	0.867 (0.826, 0.911)
		**P for interaction**						**0.7378**
	**Hip fracture**	**Normal bone mineral density**	**No**	**85616**	**813**	**669355.50**	**1.21**	**1.00 (reference)**
			Yes	28003	205	217990.15	0.94	0.802 (0.688, 0.934)
		Osteopenia	No	179814	2093	1412087.72	1.48	1.00 (reference)
			Yes	54842	547	427736.31	1.28	0.882 (0.803, 0.970)
		Osteoporosis	No	153156	2185	1201814.85	1.82	1.00 (reference)

Lifestyle factor	Fracture outcome	Bone mineral density category	Category	Participants, n	Fracture events, n	Follow-up duration, person-years	Incidence rate per 1,000 person-years	Adjusted hazard ratio (95% CI)
			Yes	40339	503	315248.47	1.60	0.890 (0.808, 0.981)
		**P for interaction**						**0.4983**
	**Other fractures**	**Normal bone mineral density**	**No**	**85616**	**6255**	**645607.60**	**9.69**	**1.00 (reference)**
			Yes	28003	2127	209954.85	10.13	1.044 (0.994, 1.097)
		Osteopenia	No	179814	16516	1347194.19	12.26	1.00 (reference)
			Yes	54842	5119	407643.38	12.56	1.022 (0.991, 1.055)
		Osteoporosis	No	153156	15579	1141436.41	13.65	1.00 (reference)
			Yes	40339	4218	298282.25	14.14	1.034 (1.000, 1.070)
		**P for interaction**						**0.7441**

Data are presented as number of participants, number of fracture events, follow-up duration in person-years, incidence rate per 1,000 person-years, and adjusted hazard ratio (95% confidence interval).
For smoking status, never smokers served as the reference group. For alcohol consumption and regular exercise, the “No” group served as the reference group.
Adjusted hazard ratios were estimated using Cox proportional hazards regression models and adjusted for income status, diabetes mellitus, dyslipidemia, hypertension, chronic kidney disease, and body mass index.
P for interaction was calculated to evaluate whether the association between each lifestyle component and fracture risk differed by BMD category.

Supplementary Table S3. Sensitivity analysis of the association between lifestyle score and fracture outcomes using lifestyle score 1 as the reference category

Fracture outcome	BMD category	Lifestyle score	Participants, n	Fracture events, n	Follow-up duration, person-years	Incidence rate per 1,000 person-years	Adjusted HR (95% CI)
**Any fracture**	**Normal BMD**	**0**	**407**	**50**	**2,886.30**	**17.32**	**1.285 (0.965, 1.709)**
		**1**	**8,066**	**795**	**59,210.91**	**13.43**	**1.00 (reference)**
		**2**	**80,287**	**7,858**	**598,461.10**	**13.13**	**0.974 (0.905, 1.048)**
		**3**	**24,859**	**2,375**	**185,123.23**	**12.83**	**0.958 (0.884, 1.038)**
	**Osteopenia**	**0**	**824**	**136**	**5,908.82**	**23.02**	**1.244 (1.046, 1.480)**
		**1**	**15,878**	**2,102**	**114,729.33**	**18.32**	**1.00 (reference)**
		**2**	**168,766**	**21,764**	**1,241,606.44**	**17.53**	**0.954 (0.912, 0.998)**
		**3**	**49,188**	**6,120**	**360,303.78**	**16.99**	**0.929 (0.884, 0.976)**
	**Osteoporosis**	**0**	**788**	**149**	**5,485.20**	**27.16**	**1.157 (0.980, 1.365)**
		**1**	**13,100**	**2,175**	**93,246.62**	**23.33**	**1.00 (reference)**
		**2**	**143,158**	**22,616**	**1,034,695.94**	**21.86**	**0.936 (0.895, 0.978)**
		**3**	**36,449**	**5,569**	**263,064.85**	**21.17**	**0.909 (0.865, 0.955)**
	**P for interaction**						**0.9543**

Fracture outcome	BMD category	Lifestyle score	Participants, n	Fracture events, n	Follow-up duration, person-years	Incidence rate per 1,000 person-years	Adjusted HR (95% CI)
Vertebral fracture	Normal BMD	0	407	15	3,045.29	4.93	1.880 (1.108, 3.190)
		1	8,066	164	61,758.02	2.66	1.00 (reference)
		2	80,287	1,607	624,345.48	2.57	0.970 (0.826, 1.139)
		3	24,859	412	193,072.37	2.13	0.822 (0.686, 0.985)
	Osteopenia	0	824	35	6,308.04	5.55	1.289 (0.916, 1.815)
		1	15,878	528	121,334.65	4.35	1.00 (reference)
		2	168,766	5,706	1,310,950.11	4.35	1.004 (0.918, 1.098)
		3	49,188	1,439	380,114.35	3.79	0.891 (0.807, 0.985)
	Osteoporosis	0	788	56	5,895.44	9.50	1.195 (0.911, 1.567)
		1	13,100	804	99,242.56	8.10	1.00 (reference)
		2	143,158	7,997	1,098,261.01	7.28	0.906 (0.842, 0.974)
		3	36,449	1,746	279,963.55	6.24	0.784 (0.721, 0.853)
Vertebral fracture	P for interaction						0.3888
Hip fracture	Normal BMD	0	407	5	3,100.41	1.61	1.260 (0.510, 3.114)
		1	8,066	77	62,191.33	1.24	1.00 (reference)
		2	80,287	749	627,990.59	1.19	0.937 (0.741, 1.185)
		3	24,859	187	194,063.32	0.96	0.773 (0.593, 1.008)
	Osteopenia	0	824	19	6,396.25	2.97	1.704 (1.064, 2.728)
		1	15,878	199	122,724.82	1.62	1.00 (reference)
		2	168,766	1,931	1,326,762.27	1.46	0.877 (0.758, 1.015)
		3	49,188	491	383,940.68	1.28	0.782 (0.663, 0.922)
	Osteoporosis	0	788	29	6,041.32	4.80	2.467 (1.669, 3.649)
		1	13,100	185	101,773.71	1.82	1.00 (reference)
		2	143,158	2,023	1,123,956.84	1.80	0.977 (0.840, 1.136)
		3	36,449	451	285,291.45	1.58	0.864 (0.728, 1.025)
Hip fracture	P for interaction						0.7140
Other fractures	Normal BMD	0	407	36	2,962.94	12.15	1.216 (0.869, 1.702)
		1	8,066	594	59,928.00	9.91	1.00 (reference)
		2	80,287	5,882	605,691.53	9.71	0.976 (0.897, 1.061)
		3	24,859	1,870	186,979.98	10.00	1.004 (0.916, 1.101)
	Osteopenia	0	824	93	6,035.82	15.41	1.186 (0.962, 1.462)
		1	15,878	1,502	117,092.35	12.83	1.00 (reference)
		2	168,766	15,491	1,265,614.82	12.24	0.951 (0.902, 1.003)

Fracture outcome	BMD category	Lifestyle score	Participants, n	Fracture events, n	Follow-up duration, person-years	Incidence rate per 1,000 person-years	Adjusted HR (95% CI)
		3	49,188	4,549	366,094.59	12.43	0.964 (0.909, 1.022)
	Osteoporosis	0	788	89	5,744.72	15.49	1.072 (0.865, 1.328)
		1	13,100	1,372	96,411.49	14.23	1.00 (reference)
		2	143,158	14,528	1,067,670.05	13.61	0.953 (0.902, 1.007)
		3	36,449	3,808	269,892.40	14.11	0.987 (0.928, 1.050)
Other fractures	P for interaction						0.9423

Data are shown as the number of participants, fracture events, follow-up duration in person-years, incidence rate per 1,000 person-years, and adjusted hazard ratio with 95% confidence interval. Cox proportional hazards models were adjusted for income status, diabetes mellitus, dyslipidemia, hypertension, chronic kidney disease, and body mass index. Lifestyle score 1 served as the reference category within each bone mineral density category. P for interaction was calculated to assess whether the association between lifestyle score and fracture risk differed by bone mineral density category.

Supplementary Table S4. Sensitivity analysis of the association between lifestyle score and fracture outcomes using a 3-year lag period

Fracture outcome	BMD category	Lifestyle score	Participants, n	Fracture events, n	Follow-up duration, person-years	Incidence rate per 1,000 person-years	Adjusted HR (95% CI)
Any fracture	Normal BMD	0	387	32	2,090.24	15.31	1.00 (reference)
		1	7,815	594	43,334.95	13.71	0.902 (0.632, 1.287)
		2	77,987	5,996	440,083.95	13.62	0.893 (0.631, 1.264)
		3	24,242	1,862	136,002.78	13.69	0.905 (0.638, 1.283)
	Osteopenia	0	780	98	4,306.85	22.75	1.00 (reference)
		1	15,239	1,541	83,605.23	18.43	0.819 (0.668, 1.005)
		2	162,459	16,376	910,378.47	17.99	0.798 (0.654, 0.973)
		3	47,459	4,617	263,626.16	17.51	0.781 (0.640, 0.955)
	Osteoporosis	0	738	109	3,953.90	27.57	1.00 (reference)
		1	12,472	1,619	67,674.47	23.92	0.877 (0.722, 1.064)
		2	136,569	16,970	754,913.55	22.48	0.824 (0.682, 0.994)
		3	34,835	4,137	191,779.76	21.57	0.793 (0.656, 0.959)
	P for interaction						0.5461
Vertebral fracture	Normal BMD	0	387	9	2,175.64	4.14	1.00 (reference)
		1	7,815	132	44,739.52	2.95	0.704 (0.359, 1.384)
		2	77,987	1,265	455,265.87	2.78	0.664 (0.345, 1.279)
		3	24,242	335	140,903.16	2.38	0.580 (0.299, 1.125)
	Osteopenia	0	780	25	4,486.02	5.57	1.00 (reference)
		1	15,239	391	87,216.14	4.48	0.793 (0.530, 1.189)
		2	162,459	4,480	949,784.52	4.72	0.838 (0.566, 1.242)
		3	47,459	1,139	274,967.16	4.14	0.751 (0.505, 1.116)
	Osteoporosis	0	738	40	4,196.85	9.53	1.00 (reference)

Fracture outcome	BMD category	Lifestyle score	Participants, n	Fracture events, n	Follow-up duration, person-years	Incidence rate per 1,000 person-years	Adjusted HR (95% CI)
		1	12,472	626	70,984.16	8.82	0.905 (0.658, 1.246)
		2	136,569	6,110	791,363.43	7.72	0.799 (0.585, 1.090)
		3	34,835	1,366	200,868.93	6.80	0.711 (0.519, 0.974)
Vertebral fracture	P for interaction						0.2498
Hip fracture	Normal BMD	0	387	4	2,199.02	1.82	1.00 (reference)
		1	7,815	61	45,035.95	1.35	0.778 (0.283, 2.141)
		2	77,987	636	457,422.34	1.39	0.781 (0.292, 2.087)
		3	24,242	164	141,532.83	1.16	0.664 (0.246, 1.792)
	Osteopenia	0	780	17	4,530.67	3.75	1.00 (reference)
		1	15,239	160	87,920.59	1.82	0.520 (0.315, 0.857)
		2	162,459	1,593	959,681.75	1.66	0.465 (0.288, 0.750)
		3	47,459	406	277,316.71	1.46	0.417 (0.256, 0.677)
	Osteoporosis	0	738	24	4,259.27	5.63	1.00 (reference)
		1	12,472	150	72,521.93	2.07	0.394 (0.256, 0.607)
		2	136,569	1,605	806,847.52	1.99	0.375 (0.251, 0.561)
		3	34,835	358	204,227.12	1.75	0.333 (0.221, 0.504)
Hip fracture	P for interaction						0.8468
Other fractures	Normal BMD	0	387	23	2,130.25	10.80	1.00 (reference)
		1	7,815	428	43,814.86	9.77	0.914 (0.601, 1.391)
		2	77,987	4,347	445,039.29	9.77	0.911 (0.605, 1.372)
		3	24,242	1,433	137,326.70	10.44	0.974 (0.645, 1.470)
	Osteopenia	0	780	62	4,388.62	14.13	1.00 (reference)
		1	15,239	1,076	84,979.10	12.66	0.911 (0.705, 1.177)
		2	162,459	11,235	926,298.21	12.13	0.870 (0.678, 1.117)
		3	47,459	3,309	267,485.60	12.37	0.887 (0.690, 1.140)
	Osteoporosis	0	738	60	4,127.30	14.54	1.00 (reference)
		1	12,472	969	69,718.59	13.90	0.974 (0.751, 1.265)
		2	136,569	10,489	776,259.77	13.51	0.945 (0.733, 1.218)
		3	34,835	2,679	196,409.06	13.64	0.954 (0.739, 1.232)
Other fractures	P for interaction						0.7471

Data are shown as the number of participants, fracture events, follow-up duration in person-years, incidence rate per 1,000 person-years, and adjusted hazard ratio with 95% confidence interval. Participants with fracture events during the first 3 years after screening were excluded. Cox proportional hazards models were adjusted for income status, diabetes mellitus, dyslipidemia, hypertension, chronic kidney disease, and body mass index. Lifestyle score 0 served as the reference category within each BMD category. P for interaction was calculated to assess whether the association between lifestyle score and fracture risk differed by BMD category.

Supplementary Table S5. Sensitivity analysis after excluding former smokers

Fracture outcome	BMD category	Lifestyle score	Participants, n	Fracture events, n	Follow-up duration, person-years	Incidence rate per 1,000 person-years	Adjusted HR (95% CI)
Any fracture	Normal BMD	0	407	50	2,886.30	17.32	1.00 (reference)
		1	7,793	766	57,269.42	13.38	0.775 (0.582, 1.032)
		2	79,407	7,778	592,087.28	13.14	0.758 (0.574, 1.001)
		3	24,632	2,347	183,472.94	12.79	0.743 (0.562, 0.984)
	Osteopenia	0	824	136	5,908.82	23.02	1.00 (reference)
		1	15,381	2,034	111,183.05	18.29	0.802 (0.674, 0.954)
		2	167,102	21,526	1,229,711.52	17.50	0.765 (0.647, 0.906)
		3	48,771	6,058	357,281.45	16.96	0.745 (0.629, 0.883)
	Osteoporosis	0	788	149	5,485.20	27.16	1.00 (reference)
		1	12,782	2,120	91,106.52	23.27	0.862 (0.731, 1.018)
		2	141,888	22,394	1,025,877.02	21.83	0.808 (0.688, 0.949)
		3	36,150	5,507	260,979.48	21.10	0.783 (0.665, 0.921)
	P for interaction						0.9527
Vertebral fracture	Normal BMD	0	407	15	3,045.29	4.93	1.00 (reference)
		1	7,793	159	59,711.47	2.66	0.533 (0.314, 0.905)
		2	79,407	1,590	617,738.68	2.57	0.516 (0.310, 0.858)
		3	24,632	405	191,328.68	2.12	0.434 (0.259, 0.726)
	Osteopenia	0	824	35	6,308.04	5.55	1.00 (reference)
		1	15,381	510	117,573.50	4.34	0.773 (0.548, 1.088)
		2	167,102	5,647	1,298,280.20	4.35	0.778 (0.558, 1.085)
		3	48,771	1,420	376,926.24	3.77	0.688 (0.492, 0.962)
	Osteoporosis	0	788	56	5,895.44	9.50	1.00 (reference)
		1	12,782	783	96,941.63	8.08	0.834 (0.636, 1.093)
		2	141,888	7,915	1,088,789.76	7.27	0.756 (0.582, 0.984)
		3	36,150	1,723	277,700.93	6.20	0.653 (0.500, 0.852)
Vertebral fracture	P for interaction						0.3934
Hip fracture	Normal BMD	0	407	5	3,100.41	1.61	1.00 (reference)
		1	7,793	73	60,147.91	1.21	0.777 (0.314, 1.924)
		2	79,407	740	621,348.61	1.19	0.742 (0.308, 1.788)
		3	24,632	183	192,318.57	0.95	0.605 (0.249, 1.470)
	Osteopenia	0	824	19	6,396.25	2.97	1.00 (reference)
		1	15,381	187	118,938.83	1.57	0.569 (0.355, 0.913)

Fracture outcome	BMD category	Lifestyle score	Participants, n	Fracture events, n	Follow-up duration, person-years	Incidence rate per 1,000 person-years	Adjusted HR (95% CI)
		2	167,102	1,901	1,313,942.25	1.45	0.511 (0.325, 0.803)
		3	48,771	486	380,703.87	1.28	0.458 (0.289, 0.724)
	Osteoporosis	0	788	29	6,041.32	4.80	1.00 (reference)
		1	12,782	182	99,407.24	1.83	0.408 (0.276, 0.604)
		2	141,888	1,995	1,114,261.94	1.79	0.393 (0.273, 0.568)
		3	36,150	443	282,968.41	1.57	0.346 (0.238, 0.504)
Hip fracture	P for interaction						0.7619
Other fractures	Normal BMD	0	407	36	2,962.94	12.15	1.00 (reference)
		1	7,793	571	57,961.07	9.85	0.818 (0.584, 1.145)
		2	79,407	5,822	599,250.57	9.72	0.803 (0.578, 1.114)
		3	24,632	1,850	185,312.42	9.98	0.824 (0.592, 1.146)
	Osteopenia	0	824	93	6,035.82	15.41	1.00 (reference)
		1	15,381	1,457	113,461.98	12.84	0.844 (0.685, 1.041)
		2	167,102	15,320	1,253,451.59	12.22	0.801 (0.653, 0.982)
		3	48,771	4,505	363,009.74	12.41	0.812 (0.661, 0.997)
	Osteoporosis	0	788	89	5,744.72	15.49	1.00 (reference)
		1	12,782	1,338	94,184.82	14.21	0.931 (0.752, 1.154)
		2	141,888	14,392	1,058,470.07	13.60	0.888 (0.721, 1.094)
		3	36,150	3,769	267,721.44	14.08	0.919 (0.745, 1.134)
Other fractures	P for interaction						0.9465

Data are shown as the number of participants, fracture events, follow-up duration in person-years, incidence rate per 1,000 person-years, and adjusted hazard ratio with 95% confidence interval. Former smokers were excluded and the lifestyle score was recalculated before repeating the primary analyses. Cox proportional hazards models were adjusted for income status, diabetes mellitus, dyslipidemia, hypertension, chronic kidney disease, and body mass index. Lifestyle score 0 served as the reference category within each BMD category. P for interaction was calculated to assess whether the association between lifestyle score and fracture risk differed by BMD category.

Supplementary Table S6. All-cause mortality rates according to bone mineral density category and lifestyle score

Grouping	Category	Participants, n	Deaths, n	Follow-up duration, person-years	Mortality rate per 1,000 person-years
Overall	All participants	541,770	22,160	4,264,133.12	5.20
BMD category	Normal BMD	113,619	4,475	890,478.20	5.03
BMD category	Osteopenia	234,656	9,131	1,847,995.26	4.94
BMD category	Osteoporosis	193,495	8,554	1,525,659.66	5.61
Lifestyle score	0	2,019	161	15,700.82	10.25
Lifestyle score	1	37,044	1,614	288,140.28	5.60
Lifestyle score	2	392,211	16,582	3,093,602.83	5.36
Lifestyle score	3	110,496	3,803	866,689.19	4.39

Mortality data are presented descriptively as the number of deaths, follow-up duration in person-years, and mortality rate per 1,000 person-years. BMD, bone mineral density.

Supplementary Table S7. Exploratory comparison of model fit and discrimination between the composite lifestyle score and individual lifestyle components

Model specification	AIC	Harrell's C-index
Composite lifestyle score	1,843,758.6	0.5563
Exercise, smoking, and alcohol entered separately	1,846,129.7	0.5211

Lower AIC indicates better relative model fit, whereas a higher C-index indicates greater discrimination. This comparison was exploratory and was not intended to establish clinical predictive superiority. AIC, Akaike information criterion; C-index, concordance index.

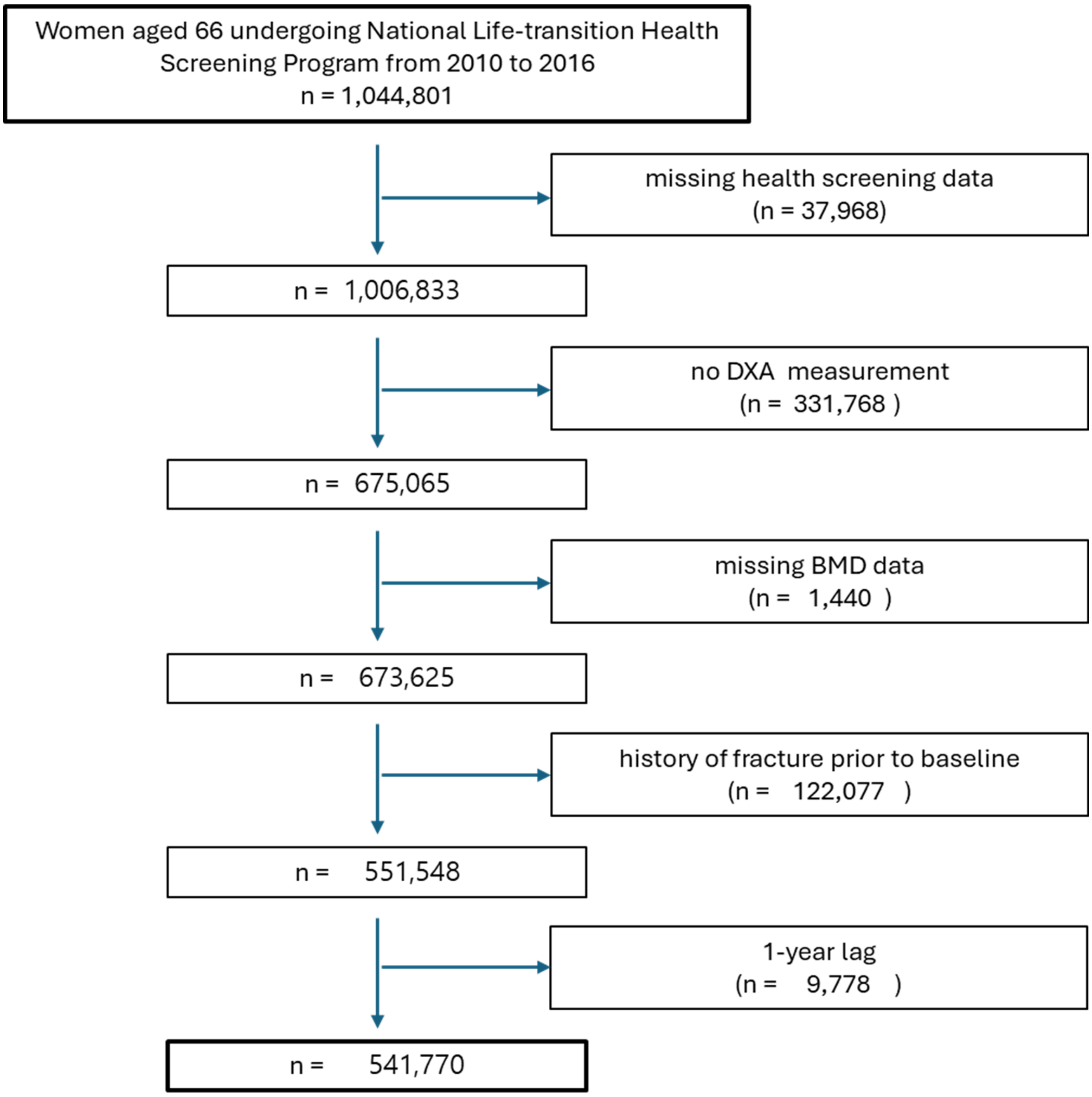

Supplementary Figure S1. Flowchart illustrating the selection process for the study population. This flowchart outlines the selection of participants utilizing data from the Korean National Health Insurance Service (NHIS).

Abbreviations: DXA, dual-energy X-ray absorptiometry; BMD, bone mineral density.

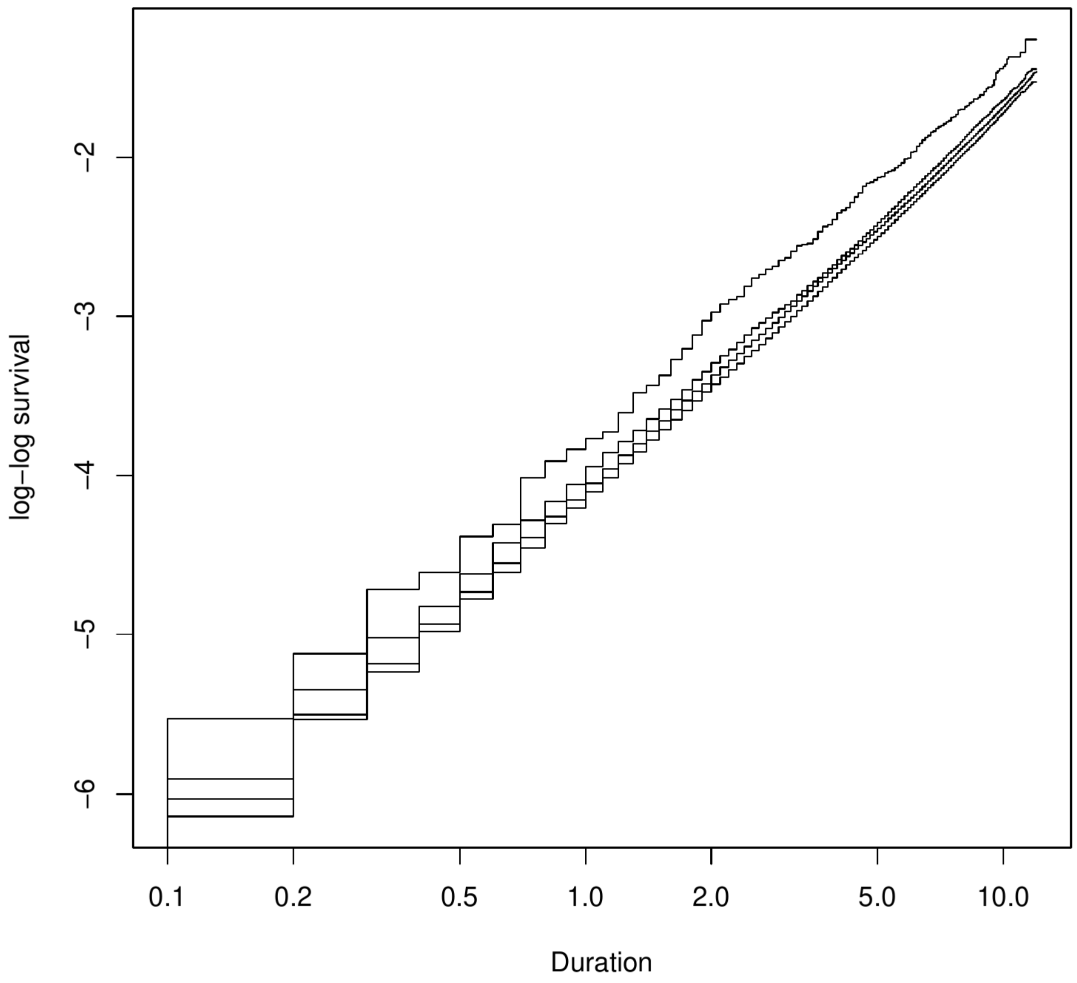

Supplementary Figure S2. Available log-log survival plot for visual assessment of the proportional hazards assumption. The trajectories were approximately parallel over most of follow-up and showed no clear indication of a major violation in the displayed comparison.
